# The Impact of m1A Methylation Modification Patterns on Tumor Immune Microenvironment and Prognosis in Oral Squamous Cell Carcinoma

**DOI:** 10.3390/ijms221910302

**Published:** 2021-09-24

**Authors:** Li Gao, Ru Chen, Masahiro Sugimoto, Masanobu Mizuta, Yo Kishimoto, Koichi Omori

**Affiliations:** 1 Department of Otolaryngology-Head and Neck Surgery, Graduate School of Medicine, Kyoto University, Kyoto 606-8507, Japan; l_gao@ent.kuhp.kyoto-u.ac.jp (L.G.); m_mizuta@ent.kuhp.kyoto-u.ac.jp (M.M.); omori@ent.kuhp.kyoto-u.ac.jp (K.O.); 2 Matsusaka City Hospital, Matsusaka 515-8544, Japan; reharrchen@gmail.com; 3 Center for Minimally Invasive Therapies, Institute of Medical Science Research and Development, Tokyo Medical University, Tokyo 160-8402, Japan; mshrsgmt@gmail.com

**Keywords:** N1-methyladenosine (m1A) modification, oral squamous cell carcinoma, tumor immune microenvironment, prognosis

## Abstract

N1-methyladenosine (m1A) modification widely participates in the occurrence and progression of numerous diseases. Nevertheless, the potential roles of m1A in the tumor immune microenvironment (TIME) are still not fully understood. Based on 10 m1A methylation regulators, we comprehensively explored the m1A modification patterns in 502 patients with oral squamous cell carcinoma (OSCC). The m1A modification patterns were correlated with TIME characteristics and the m1A score was established to evaluate the effect of the m1A modification patterns on individual OSCC patients. The TIME characteristics and survival outcomes under the three m1A modification patterns were significantly distinct. OSCC patients in the high m1A score group were characterized by poorer prognosis, lower immune infiltration, lower ssGSEA score, lower expression levels of immune checkpoint molecules, and higher tumor mutation loads. The present study revealed that m1A modification might be associated with the TIME in OSCC, and has potential predictive ability for the prognosis of OSCC.

## 1. Introduction

Oral squamous cell carcinoma (OSCC) accounts for approximately 90% of all oral cancers [1]. OSCC is characterized by high neck lymph node metastasis rates, high recurrence, and poor prognosis; it severely affects the life quality of patients worldwide [2]. The risk factors associated with OSCC include smoking, alcohol consumption, and betel quid chewing [3,4]. Although the therapeutic strategies (such as surgery, chemotherapy, and radiation) have greatly improved during the past few decades, the prognosis of OSCC has not improved significantly [5,6]. For those patients with neck lymph node metastasis, the 5-year survival rate was only 45% to 50% [7]. Thus, in-depth studies are needed to explain the potential mechanisms involved in the pathological process of OSCC and to design more effective therapeutic approaches.

Immunotherapy, including immune checkpoint inhibitor (ICI) therapy, is a novel tumor therapeutic strategy aimed at strengthening the immune system to regain the control over tumor growth and facilitate personalized treatment of cancers [8]. In recent years, ICIs, such as cytotoxic T lymphocyte-associated antigen 4 (*CTLA-4*) and programmed cell death protein 1 (*PD-1*)/PD ligand 1 (*PD-L1*) blockades, have been frequently applied in clinical oncotherapy. Inspiring therapeutic outcomes, such as improved overall survival (OS) and tumor shrinkage, have been verified in the head and neck squamous cell carcinoma (HNSCC) [9,10]. However, only a few patients with HNSCC/OSCC can benefit from ICI therapy. For the majority of cases, the clinical outcome of using ICIs is poor, and an immunosuppressed microenvironment and a limited reinvigoration of antitumor immunity are largely to blame [11,12]. Further exploration of the regulatory mechanism of the tumor immune microenvironment (TIME) is crucial for predicting the effect of ICI therapy and designing optimal personalized therapeutic strategies.

Growing evidence suggests RNA chemical modifications have important functions in fundamental cellular processes such as cellular differentiation, protein production, cell signaling, and maintenance of the circadian rhythm [13,14,15]. RNA methylation is one of the most common patterns of RNA chemical modification observed during the epigenetic modification of posttranscriptional RNA, including N1-methyladenosine (m1A), N3-methylcytosine (m3C), 5-methylcytosine (m5C), and N6-methyladenosine (m6A). m1A is ubiquitous in tRNA, rRNA, mRNA, and mitochondrial transcripts [16]. The majority of m1A is found in the GC-rich sequence with highly structured 5′-untranslated regions (UTRs) near the translation initiation site of mRNA. It has been verified that m1A dysregulation affects multiple cellular processes, including RNA structural stability, folding, interactions with proteins, cell viability, impaired self-renewal ability, cell proliferation, and cell death [17,18].

The regulators of m1A methylation include “writers” (*TRMT10C*, *TRMT61B*, and *TRMT6/61A*), “readers” (*YTHDF1-3* and *YTHDC1*), and “erasers” (*ALKBH1* and *ALKBH3*) [16,19,20]. Generally, the status of m1A is mediated by “writers” and “erasers”, while “readers” mediate m1A-related functions. The “writers” act as methyltransferase complexes. *TRMT61B* and *TRMT6/61A* catalyze m1A modification at position 58 of mt and cyt tRNA in human cells; *TRMT10C* does so at position 9 [21,22,23]. *ALKBH3* and *ALKBH1* are AlkB family proteins. As “erasers”, they serve as m1A demethylases [24]. The “readers” mediate the translation and degradation of downstream RNA. *YTHDF1-3* and *YTHDC1* belong to YTH domain-containing proteins and directly bind to the reading frame of RNA with m1A [25].

Recent studies have indicated that m1A modification widely participates in the occurrence and progression of many diseases [26,27,28]. *ALKBH3* is reported to be highly expressed in a number of human cancers; knockdown of *ALKBH3* increased m1A levels in tRNA and decreased protein synthesis in cancer cells [29]. Pilžys et al. have reported that *ALKBH3* and *ALKBH1* are overexpressed in HNSCC [30]. In addition, the frameshift mutation in repeat sequences of *TRMT6* has been demonstrated in colon cancer [31]. However, the above studies only focused on one or two m1A regulators, while the progression of cancers depends on the interaction between multiple m1A methylation regulators. Hence, a comprehensive evaluation of multiple m1A methylation regulators will enrich our knowledge of the oncogenesis and tumor progression in OSCC.

In the present study, we analyzed the m1A modification patterns in 502 patients with OSCC and correlated m1A modification patterns with TIME characteristics. Three distinct m1A modification patterns were identified. The TIME characteristics and survival outcomes under these m1A modification patterns were distinct, which indicated m1A modification may significantly influence the formation of individual TIME in OSCC patients. We further established m1A gene signatures and m1A score signatures to quantify m1A modification patterns in individual OSCC patients.

## 2. Results

### 2.1. Genetic Variation and Prognostic Relevance of m1A RNA Methylation Regulators in OSCC

**Figure 1:**
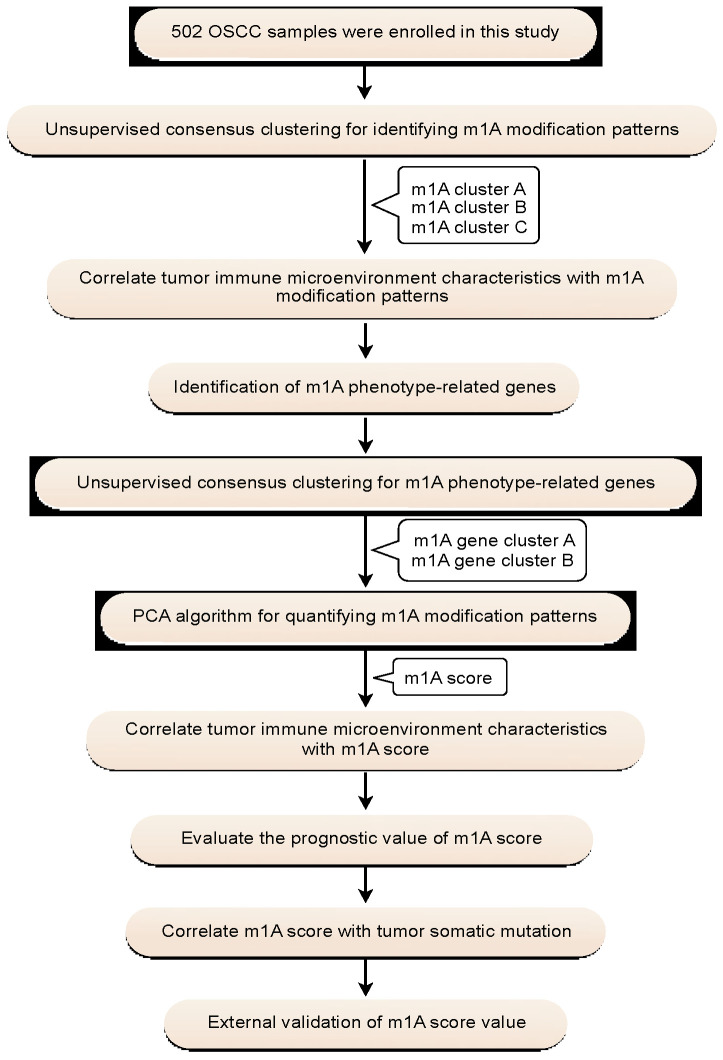
Flowchart of the study design.

**Figure 2:**
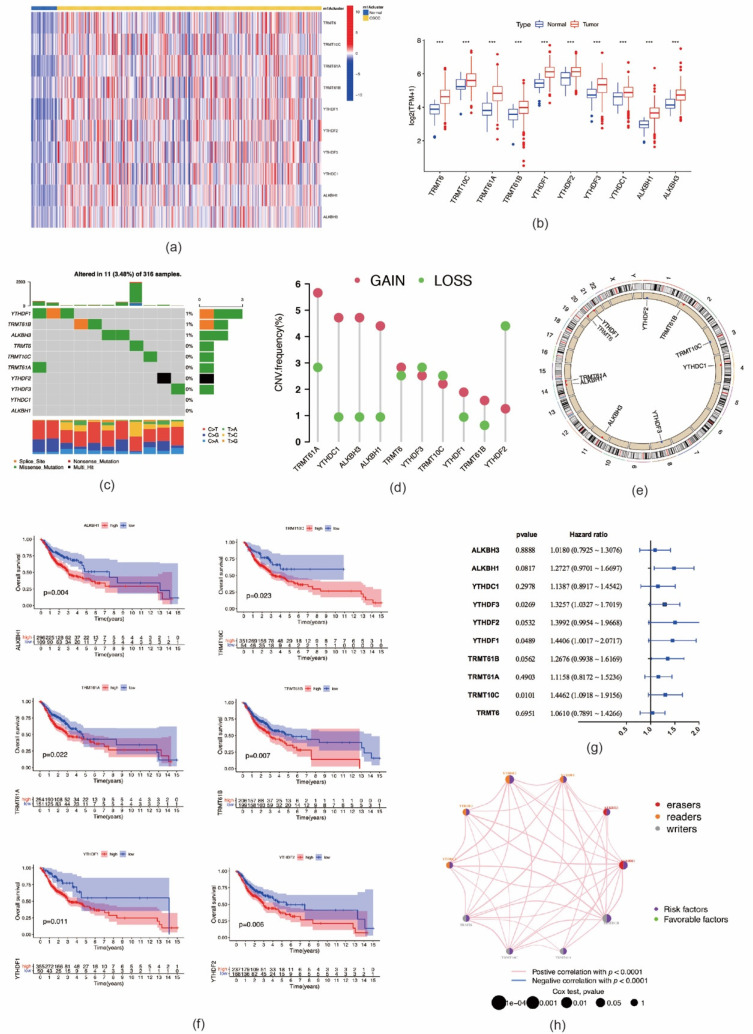
Genetic variation and prognostic relevance of m1A regulators in oral squamous cell carcinoma. (**a**) The expression levels of 10 m1A methylation modification regulators in each clinical sample. (**b**) The expression of 10 m1A methylation regulators between tumor tissues and normal controls (*** *p* < 0.001). (**c**) 11 of 316 samples (3.48%) from TCGA–HNSCC cohort show genetic alterations of 10 m1A regulators. (**d**) The CNV mutation frequency of m1A regulators in TCGA–HNSCC cohort. (**e**) The location of CNV alteration of m1A regulators on chromosomes. (**f**) Survival analysis of m1A regulators in patients with oral squamous cell carcinoma. (**g**) The prognostic analyses for 10 m1A regulators using univariate Cox regression model. (**h**) The interaction between m1A regulators in oral squamous cell carcinoma. The left part of the circle represents the category of each regulator. Erasers, red; readers, orange; writers, gray. The right part of the circle represents the survival impact of each regulator. Favorable factors of prognosis, green; risk factors of prognosis, purple.

A flowchart of the study design is shown in Figure 1. In this study, 10 m1A regulators were analyzed, including “writers” (*TRMT10C*, *TRMT61B*, and *TRMT6/61A*), “readers” (*YTHDF1-3* and *YTHDC1*), and “erasers” (*ALKBH1* and *ALKBH3*). Compared with normal controls, all m1A regulators in OSCC samples were markedly upregulated (*p* < 0.001) (Figure 2a,b). Somatic mutation analysis showed several mutation types were involved, such as splice site mutations, nonsense mutations, missense mutations, and multiple hits. Among the m1A regulators, *YTHDF1*, *TRMT61B*, and *ALKBH3* showed a relatively higher frequency of somatic mutations. However, the mutation frequency was relatively low, with only 11 of 316 samples (3.48%) showing m1A regulator mutations (Figure 2c). CNV analysis demonstrated that CNV alterations were prevalent. Among these m1A regulators, *TRMT61A*, *YTHDC1*, *ALKBH3*, *ALKBH1*, *YTHDF1*, and *TRMT61B* showed higher copy number gain, while *YTHDF2* showed higher copy number loss (Figure 2d,e). These results indicated that expressional and genetic variations in m1A regulators were distinct between OSCC and normal tissue samples.

We further analyzed the prognostic value of these m1A regulators in OSCC. Kaplan–Meier analysis showed that six m1A regulators were associated with the prognosis of OSCC patients. The high expression levels of *ALKBH1* (*p* = 0.004), *TRMT10C* (*p* = 0.023), *TRMT61A* (*p* = 0.022), *TRMT61B* (*p* = 0.007), *YTHDF1* (*p* = 0.011), and *YTHDF2* (*p* = 0.006) were associated with significantly shorter OS (Figure 2f). In the validation cohort, high expression of the following nine m1A regulators was associated with worse OS outcomes: *TRMT61A* (*p* = 0.001), *TRMT61B* (*p* = 0.003), *TRMT10C* (*p* = 0.026), *TRMT6* (*p* = 0.005), *YTHDF1* (*p* = 0.047), *YTHDF2* (*p* = 0.006), *YTHDF3* (*p* < 0.001), *ALKBH1* (*p* < 0.001), and *ALKBH1* (*p* = 0.013) (Figure S1). The interactions of m1A regulators and their prognostic value in OSCC are presented in Figure 2g,h. All m1A regulators exhibited high positive correlations with each other. In addition, these m1A regulators demonstrated tumor-promoting effects; i.e., high expression of m1A regulators suggested poor prognosis of patients with OSCC. The above-mentioned results imply that m1A regulators might affect the prognosis of OSCC patients.

### 2.2. Consensus Clustering of m1A Regulators in Three Clusters Correlated with OSCC Prognosis and Immune Microenvironment

**Figure 3:**
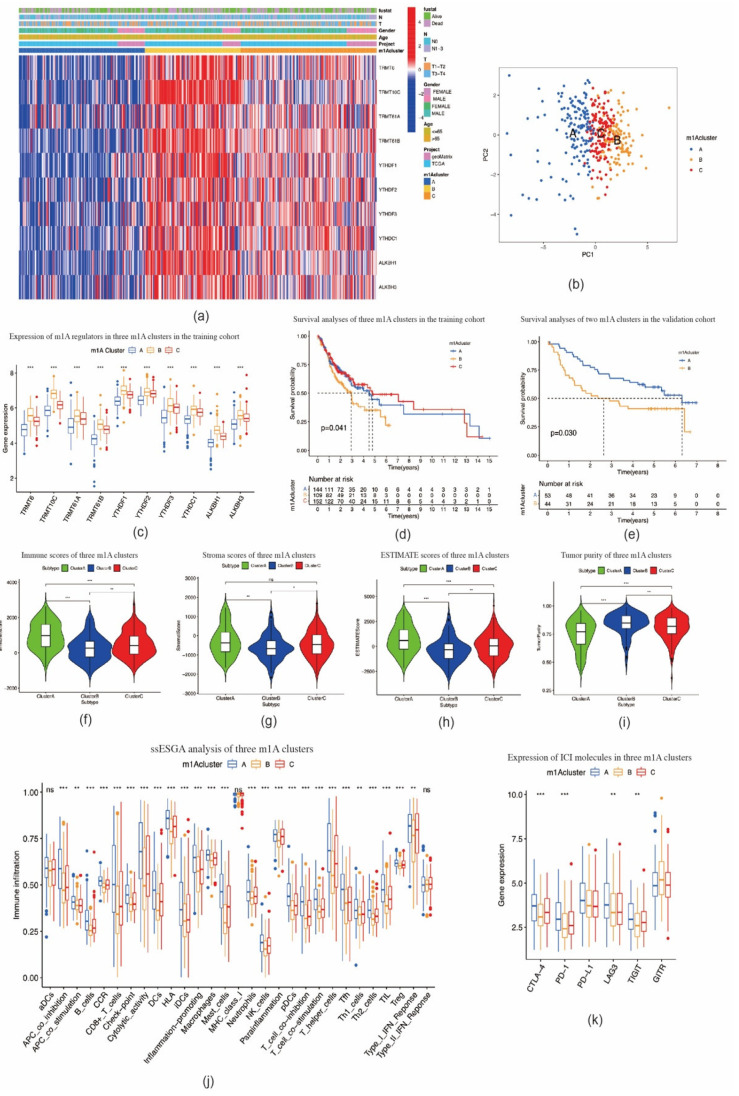
TIME–related characteristics of each m1A modification pattern. (**a**) Heat map of consensus clustering of 10 m1A regulators in oral squamous cell carcinoma. (**b**) Principal component analysis shows three m1A modification patterns could be clearly distinguished. (**c**) The expression of 10 m1A methylation regulators among m1A modification patterns. (**d**) The overall survival for 405 patients with oral squamous cell carcinoma within different m1A modification clusters (*p* = 0.041, log–rank test). (**e**) m1A modification clusters in the validation cohort were also significantly related to overall survival (*p* = 0.030, log–rank test). (**f**–**i**) Violin plots showing the immune, stroma, and ESTIMATE scores and tumor purity in three m1A modification clusters. (**j**) The abundance of each TIME–infiltrating cell in three m1A modification patterns. (**k**) The expression of immune checkpoint molecules in three m1A modification patterns. * *p* < 0.05; ** *p* < 0.01; *** *p* < 0.001; ns = not significant.

Based on the expression levels of m1A regulators, consensus clustering analysis was performed. K = 3 was deemed to be the most optimal selection for dividing OSCC patients into the following three clusters: cluster A (*n* = 144), cluster B (*n* = 109), and cluster C (*n* = 153) (Figure S2a–g). The expression pattern of m1A regulators in these three clusters and related clinicopathological characteristics are depicted in a heat map (Figure 3a). The PCA results showed that the three clusters could be clearly distinguished (Figure 3b). The expression levels of m1A regulators in cluster B were noticeably higher than those in clusters A and C (Figure 3c). Furthermore, Kaplan–Meier analysis showed a significantly shorter OS of patients with OSCC in cluster B than of those in clusters A and C (*p* = 0.041) (Figure 3d). In the validation cohort, OSCC patients were divided into two clusters (Figure S2h–n). High expression of m1A regulators also suggested poor prognosis of patients with OSCC (*p* = 0.03) (Figure 3e, Figure S2o), which indicates the reliability and stability of our findings.

**Figure 4:**
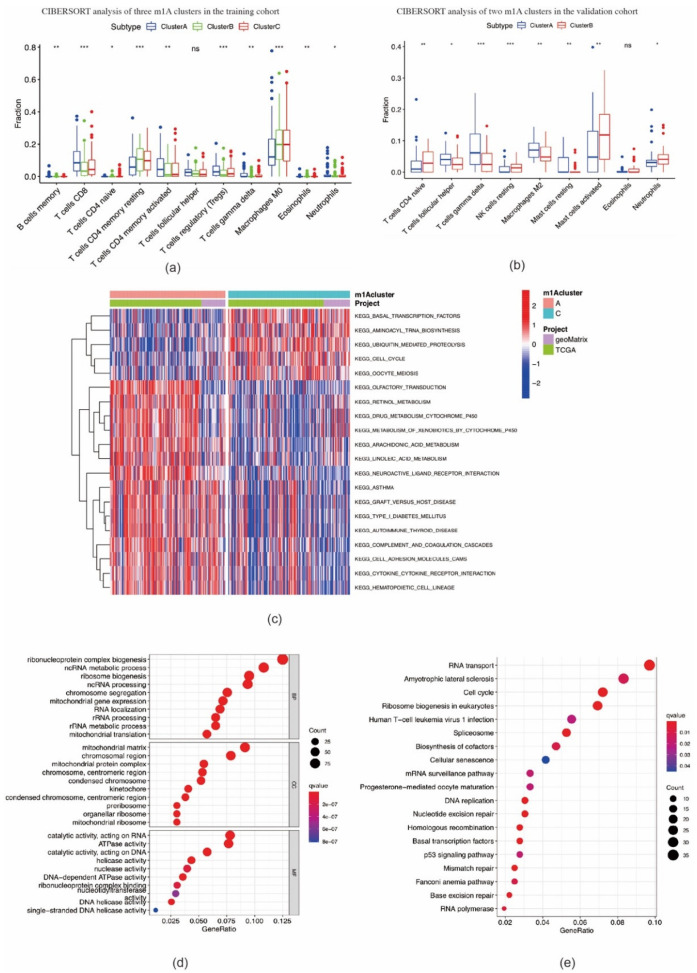
CIBERSORT analysis of m1A modification patterns and biological characteristics of m1A phenotype–related genes. (**a**,**b**) The fraction of tumor–infiltrating lymphocyte cells in m1A modification patterns in training cohort (**a**) and validation cohort (**b**) using the CIBERSORT algorithm (* *p* < 0.05; ** *p* < 0.01; *** *p* < 0.001; ns = not significant). (**c**) The activation states of biological pathways between clusters A and C using GSVA enrichment analysis. (**d**,**e**) Functional annotation for overlapping m1A phenotype–related genes using GO enrichment analysis (**d**) and KEGG enrichment analysis (**e**).

We then investigated the correlation between the three m1A clusters and TIME in OSCC. Immune cell infiltration analysis results demonstrated that the immune, stromal, and ESTIMATE scores in cluster B were markedly lower, whereas the tumor purity score was markedly higher than those in clusters A and C (Figure 3f–i). The ssGSEA analysis indicated that the enrichment scores for most immune cell types (26 groups among all 29 groups), including tumor-infiltrating lymphocytes (TILs), dendritic cells (DCs), and T cells, were significantly different among m1A clusters A–C. Among these three clusters, cluster B had the lowest enrichment scores (Figure 3j). In addition, the expression levels of ICI molecules *CTLA-4*, *PD-1*, *LAG3*, and *TIGIT* in cluster B were markedly lower than those observed in clusters A and C (Figure 3k). CIBERSORT analysis indicated the fraction of CD8 T cells in cluster B was lower, while the fraction of CD4 naïve T cells and CD4 memory resting T cells in cluster B was higher than those in clusters A and C (Figure 4a). In the validation cohort, the fraction of CD4 naïve T cells was also found to be higher in the cluster with a poorer prognosis (Figure 4b). GSVA enrichment analysis showed cluster A was markedly enriched in retinol metabolism, linoleic acid metabolism, and arachidonic metabolism-related pathways. Cluster B was enriched in RNA processing, cell cycle, and mismatch repair related pathways, while cluster C was enriched in chemokine signaling, leukocyte transendothelial migration, and aminoacyl tRNA biosynthesis-related pathways (Figure 4c; Figure S3a,b). These outcomes indicate that m1A-related patterns may affect the TIME in OSCC, and thus potentially influence the prognosis and effects of ICI therapy in OSCC.

### 2.3. Identification of m1A Gene Signatures in OSCC

A total of 827 m1A phenotype-related DEGs were screened out among three m1A modification patterns (Figure S4a, Table S1). As shown in Figure 4d, DEGs were found to be remarkably enriched in RNA and mitochondrial biogenesis and activity, chromosome processing, and ATPase activity based on GO analysis, and also RNA transport, cell cycle, ribosome biogenesis in eukaryotes, p53 signaling pathway, and other pathways based on KEGG analysis (Figure 4e). Enrichment results demonstrated that DEGs were markedly related to RNA processing and immune responses, suggesting that dysfunction of m1A may affect oncogenesis and tumor progression in OSCC.

**Figure 5:**
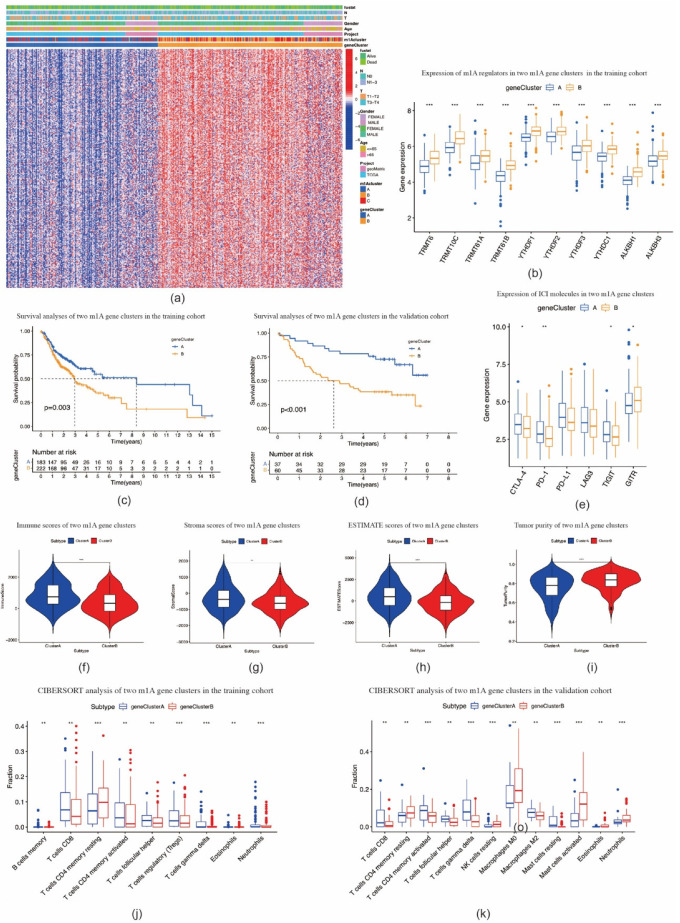
Construction of m1A gene signatures. (**a**) Heat map of consensus clustering of m1A phenotype–related genes to divide patients with oral squamous cell carcinoma into two genomic subtypes. (**b**) The expression of 10 m1A methylation regulators between two m1A modification genomic clusters. (**c**) The overall survival for 405 patients with oral squamous cell carcinoma within different m1A modification genomic clusters (*p* = 0.003, log–rank test). (**d**) The overall survival for patients with oral squamous cell carcinoma in the validation cohort within different m1A modification genomic clusters (*p* < 0.001, log–rank test). (**e**) The expression of immune checkpoint molecules in two m1A modification genomic clusters. (**f**,**i**) Violin plots show the immune, stroma, and ESTIMATE scores and tumor purity in two m1A modification genomic clusters. (**j**,**k**) The fraction of tumor–infiltrating lymphocyte cells in m1A modification genomic clusters in training cohort (**j**) and validation cohort (**k**) using the CIBERSORT algorithm. * *p* < 0.05; ** *p* < 0.01; *** *p* < 0.001.

Based on these m1A phenotype-related DEGs, we divided OSCC patients into two m1A gene clusters, namely, cluster A (*n* = 184) and cluster B (*n* = 223) (Figure S4b–h). Compared with cluster A, the expression levels of all m1A regulators in cluster B were noticeably higher (*p* < 0.001) (Figure 5a,b). The patients with OSCC in cluster B were found to be associated with poorer prognosis (*p* = 0.003) (Figure 5c). A similar OS result was obtained in the validation cohort (*p* < 0.001) (Figure 5d, Figure S4i–o). Compared with cluster A, the expression levels of *PD-1*, *CTLA-4*, *TIGIT*, and *GITR* in cluster B were significantly lower (Figure 5e). Furthermore, cluster B showed higher scores for immune, stromal, and ESTIMATE scores, and lower tumor purity scores, when compared with cluster A (Figure 5f–i). In both TCGA-HNSCC and validation cohorts, the fraction of CD8-positive T cells declined while that of CD4 memory resting T cells increased in cluster B (Figure 5j–k). These results were similar to the results in m1A methylation modification patterns.

### 2.4. Construction of the m1A Gene Signature

**Figure 6:**
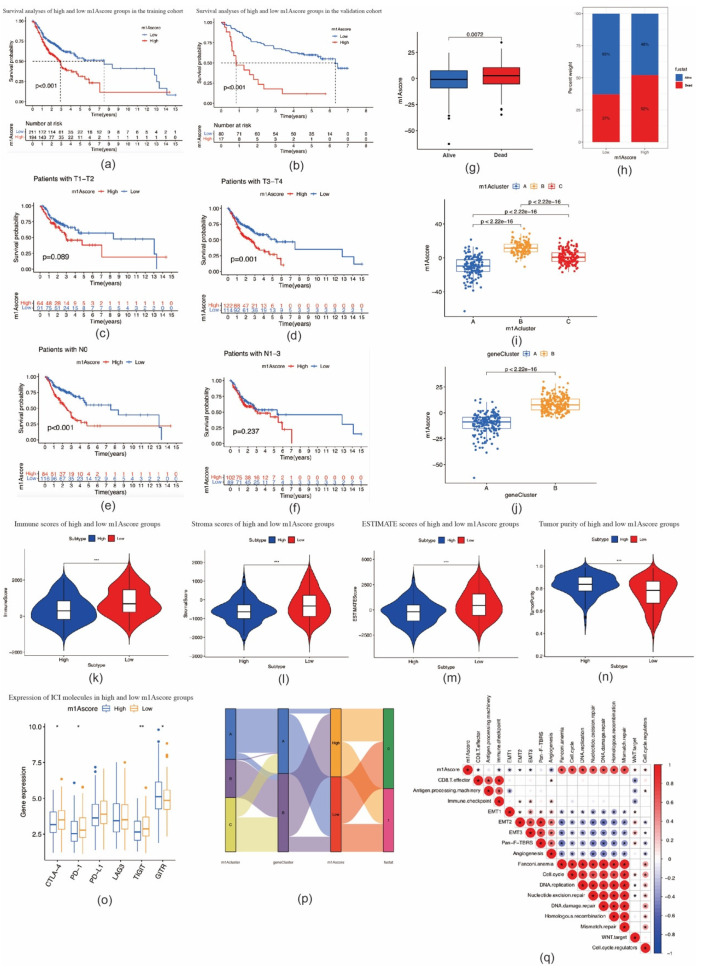
Construction of m1A score and exploration of the relevance of clinical features. (**a**) Survival analyses for patients with oral squamous cell carcinoma in the low and high m1A score groups (*p* < 0.0001, log–rank test). (**b**) Survival analyses for low and high m1A score patients in validation cohort (*p* < 0.0001, log–rank test). (**c**,**d**) Survival outcomes of low and high m1A score patients with oral squamous cell carcinoma at T1–T2 (**c**) and T3–T4 stages (**d**). (**e**,**f**) Survival outcomes of low and high m1A score patients with oral squamous cell carcinoma at N0 (e) and N1–N3 stages (**f**). (**g**) Differences in m1A scores between distinct survival status. (**h**) The proportion of survival status of patients in low or high m1A score groups. Alive/dead: 63%/37% and 48%/52% in the low and high m1A score groups, respectively. (**i**) Differences in m1A scores among three m1A modification patterns in patients with oral squamous cell carcinoma (*p* < 0.001, Kruskal–Wallis test). (**j**) Differences in m1A score between two gene clusters in patients with oral squamous cell carcinoma (*p* < 0.001, Student’s *t*-test). (**k**–**n**) Violin plots show the immune, stroma, and ESTIMATE scores and tumor purity between high and low m1A score groups. (**o**) The expression of immune checkpoint molecules between high and low m1A score groups (* *p* < 0.05; ** *p* < 0.01; *** *p* < 0.001). (**p**) Alluvial diagram showing the changes in m1A modification clusters, gene clusters, and m1A scores. (**q**) Correlations between m1A score and known biological gene signatures in patients with oral squamous cell carcinoma using Spearman analysis.

To quantify m1A modification patterns in individual OSCC patients, m1A scores were evaluated on the basis of phenotype-related genes. As shown in Figure 6a, the survival outcomes of OSCC patients in the high m1A score group were significantly worse than those in the low m1A score group (*p* < 0.001). The validation cohort also showed consistent OS results (Figure 6b). In addition, at different T and N stages, OSCC patients with a high m1A score demonstrated significant survival impairment (Figure 6c–f). Kruskal–Wallis tests confirmed OSCC patients with high m1A scores were more associated with poor survival outcomes compared to those with low m1A scores (*p* = 0.0072) (Figure 6g–h). In addition, there was a significant difference in m1A scores between the m1A clusters. m1A cluster B had the highest median score, while m1A cluster A had the lowest median score (Figure 6i). The m1A gene cluster B showed markedly increased m1A scores compared to m1A gene cluster A (Figure 6j). Immune cell infiltration analysis results and ICI molecule expression patterns were consistent with the m1A gene signature patterns (Figure 6k–o). The alluvial diagram showed that most OSCC patients in m1A cluster B were attributed to the m1A gene cluster B and were linked to a high m1A score (Figure 6p). We further analyzed the correlation between the m1A score and known biological gene signatures. m1A score was positively correlated with mismatch repair and cell cycle-related signatures, such as nucleotide excision repair, mismatch repair, DNA replication, and DNA damage repair, and negatively correlated with immune checkpoint, EMT1, angiogenesis, and CD8 T effector (Figure 6q).

**Figure 7:**
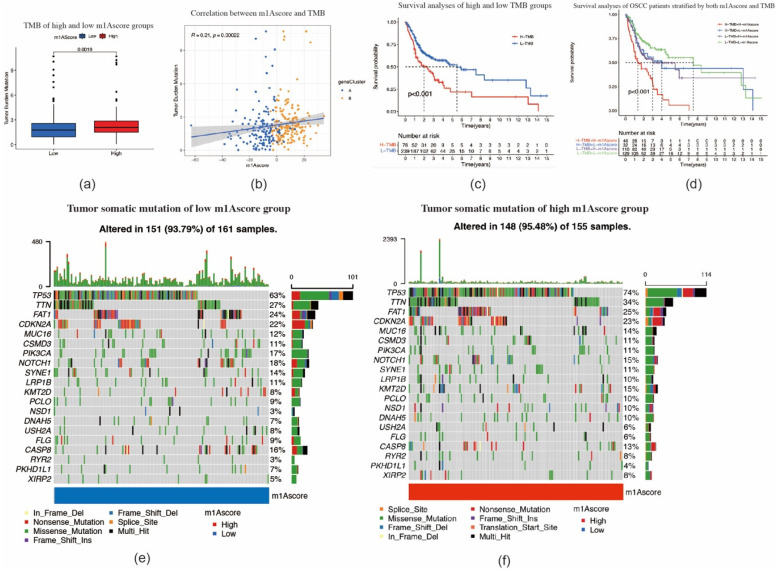
Tumor mutation–related characteristics for m1A scores. (**a**) The high m1A score tumors were markedly correlated with a higher tumor mutational burden (TMB) (*p* < 0.001, Student’s *t*-test). (**b**) The correlation between m1A score and TMB exhibits a significant positive correlation (*p* = 0.00022). (**c**) Survival analysis for patients in high and low TMB subgroup using Kaplan–Meier curves (*p* < 0.0001, log–rank test). (**d**) Survival analysis for subgroup patients stratified by both m1A score and TMB quantification. H, high; L, low. (**e**,**f**) The tumor somatic mutation landscape of significantly mutated genes in patients with oral squamous cell carcinoma stratified by low m1A score (**e**) and high m1A score (**f**).

We subsequently performed TMB quantification analyses to verify the correlation between TMB and m1A scores. As shown in Figure 7a,b, tumors with high m1A scores were significantly associated with higher TMB (*p* = 0.0019). The m1A score and TMB showed a significant positive correlation. Patients with high m1A scores had a higher proportion and more extensive tumor mutation burden than those with low m1A scores (Figure 7e,f). Furthermore, OSCC patients with high TMB had a poorer prognosis (*p* < 0.001) (Figure 7c). OSCC patients with a combination of high TMB and a high m1A score showed the worst prognosis among all patients with OSCC (*p* < 0.001) (Figure 7d). Taken together, our study indicates that the m1A gene signature is strongly correlated with the pathological progression of OSCC, and thus markedly affects the prognosis of OSCC.

## 3. Discussion

Accumulated evidence reveals that the disorders pertaining to m1A are widely linked to the occurrence and progression of many diseases [32]. On the other hand, an in-depth study of the regulatory mechanism of TIME is crucial for understanding the oncogenesis and progression of tumors. In this study, we evaluated the effect of m1A methylation regulators on the TIME of OSCC to provide insights into the TIME antitumor immune response, which would help in the development of more effective personalized immunotherapy strategies.

Here, we identified three m1A modification patterns based on 10 m1A regulators. Among these clusters, cluster B was characterized by a higher expression level of m1A regulators and poorer prognosis. Immune, stromal, and ESTIMATE scores were markedly lower and tumor purity was higher in cluster B than those in clusters A and C. Substantial studies have indicated that the crucial role of TILs in the TIME of OSCC, which could affect or predict the treatment effect of ICIs [33,34,35]. High CD8-positive T cell expression among TILs is thought to be associated with a better OS outcome in HNSCC [36]. A high CD4/CD8 ratio is correlated with a poor prognosis for cancer patients [34,37]. Our CIBERSORT analysis results validated these observations. As an immunosuppressive disease, OSCC has been shown to be associated with inactive natural killer cells (NK), T cells, dendritic cells (DCs), and TILs [38]. Our ssGSEA analysis showed that cluster B had the lowest enrichment scores, including the immune cell types mentioned above. Conversely, the enrichment scores in cluster A were the highest. To our surprise, patients in cluster A did not show a matching survival advantage, and cluster C exhibited the best prognosis among these three groups. GSVA enrichment analysis indicated that the arachidonic acid metabolism pathway was enriched in cluster A, and this pathway might be involved in the process of the progression of OSCC [39]. Recent studies have demonstrated that TILs may also lead to an immune-promoting function depending on which cell subset dominates [40,41,42]. Watanabe et al. have also indicated that the in situ balance between effector T cells and regulatory T cells is the most important factor for predicting survival in OSCC [41]. We speculated that the above-mentioned reasons may weaken the antitumor effect of TIME cells and further induce the poorer prognosis in cluster A compared to that in cluster C. Therefore, we concluded that m1A-related patterns may affect the TIME in OSCC, and thus potentially influence the prognosis of OSCC.

Based on m1A signature genes, we identified two m1A genomic clusters which were markedly associated with different prognoses and TIME characteristics. Considering the individual heterogeneity of m1A modifications, the m1A modification pattern in individual OSCC patients were analyzed using the m1A score scoring systems. Similar to the clustering results of the m1A gene clusters, the TIME characteristics between the high and low m1A score clusters were significantly different. Further analysis demonstrated that a higher m1A scores were associated with high TMB and poor survival outcomes. This suggests that m1A scores may act as a potential biomarker for determining the TIME infiltration patterns and predicting the prognosis of OSCC.

ICI therapy has become a promising therapeutic option for HNSCC/OSCC. At present, two *PD-1* blockades, namely nivolumab and pembrolizumab, have been approved by the FDA for relapsed or metastatic HNSCC patients with platinum resistance [43]. *PD-1* has been proven to contribute to the longer median OS in comparison with traditional chemotherapy in HNSCC by the following three randomized phase III trials: 8.4 months vs. 6.9 months in KEYNOTE-040; 11.6 months vs. 10.7 months in KEYNOTE-048, and 7.5 months vs. 5.1 months in CHECKMATE-141 [44,45,46]. It is generally accepted that upregulation of immune-checkpoint molecules can be induced by tumors, leading to a prominent immune evasion mechanism [47]. *CTLA-4*, *PD-1*, *PD-L1*, *LAG3*, *TIGIT*, and *GITR* have been reported as immunosuppressive molecules that help in maintaining the host tolerance by attenuating T cell functions [48]. Our study verified that the expression of *CTLA-4*, *PD-1*, and *TIGIT* is positively associated with m1A regulator expression, immune infiltration, and ssGSEA scores in m1A modification patterns, m1A gene clusters, and m1A score clusters. *PD-1* has been reported to be a potential predictive biomarker of disease response to immunotherapy, and high expression of *PD-1* is thought to be related to poor prognosis, progression, and metastasis of OSCC [49]. A recent study has indicated that *TIGIT* was highly expressed in both peripheral blood mononuclear cells and TILs from OSCC patients, and high expression of *TIGIT* in CD4 positive T cells (19.0%) and CD8-positive T cells (35.9%) showed a high correlation with the higher T stage and nodal invasion [50]. Thus, we speculate that m1A modifications could affect the therapeutic efficacy of ICIs, which may serve as a predictor of the therapeutic efficacy of ICIs.

HPV infection was considered to be one of the factors in the carcinogenesis of OSCC. However, recent clinical and fundamental studies have indicated that the effect of HPV infection on the carcinogenesis and survival rates of OSCC is rare [51]. In the present study, we used a microarray dataset containing 97 HPV-negative OSCC patients as a validation cohort. The expression level of m1A regulators, immune infiltration analysis, ssGSEA scores, CIBERSORT analysis, and the expression level of ICI molecules in the validation cohort were almost consistent with those observed in the training cohort, which indicates the reliability and stability of our findings. However, further validation of the effect of m1A methylation modification by in vitro or in vivo experiments are needed in future studies.

To summarize, the present study evaluated the correlation between m1A modifications and TIME characteristics in 502 patients with OSCC. The difference in m1A modification patterns provides a clue for understanding the impact of TIME on the carcinogenesis and progression of OSCC. The systematic evaluation of the m1A modification patterns in individual OSCC patients enrich our knowledge of TIME characterization and might help with prognosis prediction and the development of more effective personalized immunotherapy strategies in OSCC.

## 4. Materials and Methods

### 4.1. Dataset Source and Preprocessing

The mRNA expression data and clinical information of OSCC patients were obtained from the TCGA (http://cancergenome.nih.gov/ (accessed on 7 May 2021)) and GEO databases (https://www.ncbi.nlm.nih.gov/geo/ (accessed on 7 May 2021)). All data were defined as open access. Patients with incomplete survival data were removed from the subsequent analysis. The RNA-seq transcriptome data (FPKM format) of OSCC in TCGA were obtained from the TCGA HNSCC cohort, including those for 322 OSCC patients diagnosed at the sites of the alveolar ridge, hard palate, oral cavity, the base of the tongue, oral tongue, floor of the mouth, and buccal mucosa, as well as 32 normal controls. Microarray data were obtained from the GSE65858 dataset in GEO and 83 OSCC samples were selected. The level 3 HTseq-FPKM data were transformed into the transcripts per kilobase million (TPM) format and then combined with microarray data using the sva package in R to remove the batch effects. Another microarray dataset, GSE41613, containing 97 OSCC samples, served as a validation cohort. Somatic mutation expression data were retrieved from TCGA and analyzed and visualized using the maftools package in R. Copy number variations (CNVs) were acquired from the UCSC Xena program and then analyzed and visualized using the RCircos package in R.

### 4.2. Unsupervised Consensus Clustering of m1A Methylation Regulators

Based on the expression of 10 m1A regulators, consensus clustering analysis was performed using the ConsensusClusterPlus R package. OSCC patients were divided into distinct subgroups. Principal component analysis (PCA) of the m1A clusters was conducted and visualized by the limma and ggplot2 packages in R. The OS of different clusters was evaluated using Kaplan–Meier and log-rank analyses. The heat map was plotted using the the pheatmap package in R.

### 4.3. TIME Cell Infiltration Evaluation

The ESTIMATE algorithm was applied to evaluate the degree of immune cell infiltration among different clusters. The immune, stromal, and ESTIMATE scores were calculated and then the tumor purity was predicted on the basis of the algorithm. A gene set of human immune cell subtypes was retrieved from published references [52,53]. The single-sample gene set enrichment analysis (ssGSEA) was used for evaluating the relative amount and activity levels of each immune cell type in the TIME of OSCC. Next, to investigate the differences in immune cell subtypes, we performed CIBERSORT analysis to assess the proportion of infiltrated immune cells in OSCC patients with the expression profiles.

### 4.4. Gene Set Variation Analysis

To explore the differences in physiological processes based on m1A modification patterns, Gene set variation analysis (GSVA) was performed using the GSVA package in R. The “c2.cp.kegg.v7.4.symbols” gene sets for GSVA were obtained from the MSigDB database (http://www.gsea-msigdb.org/gsea/msigdb (accessed on 10 May 2021)). Adjusted *p*-values of <0.05 were considered to be statistically significant.

### 4.5. Identification of m1A-Related Differentially Expressed Genes

The identification of m1A-related differentially expressed genes (DEGs) between m1A modification patterns was based on the methods described in previous literature [6]. Based on the expression of 10 m1A regulators, OSCC patients were classified into three distinct m1A modification patterns. DEGs between the different m1A patterns were screened out using the empirical Bayesian approach in the limma package in R, and having adjusted *p*-values of <0.001 was the significance criterion for DEG screening.

### 4.6. GO and KEGG Pathway Enrichment Analyses

GO and KEGG pathway enrichment analyses of the DEGs were performed using the clusterProfiler package and the ggplot2 package in R. The cutoff criteria were set as *p* < 0.01 and *q* < 0.05.

### 4.7. Construction of m1A Gene Signature

To quantitatively analyze the effect of m1A modification patterns on individual OSCC patients, the m1A gene signature, also termed the m1A score, was evaluated. The method for m1A score establishment has previously been described by Zhang et al. [54]. Briefly, the overlapping DEGs among the different m1A clusters were screened out. OSCC patients were divided into several groups using the consensus clustering analysis for overlapping DEG analysis. Subsequently, univariate Cox regression analysis was used to assess the prognosis of each DEG. Finally, PCA analysis was conducted to establish the m1A gene signature. Principal components (PCs) 1 and 2 were extracted and selected as signature scores. We applied a formula similar to the Genomic Grade Index (GGI) to define the m1A score [54,55]:

m1Ascore = ∑(PC1_i_ +PC2_i_)

where i is the expression of m1A phenotype-related genes.

### 4.8. Statistical Analysis

Statistical analyses and visualization were performed using R software (version 3.5.3, https://www.r-project.org/), GraphPad Prism v6.0 for Mac (GraphPad; San Diego, CA, USA). The survival analyses between the groups of patients were performed using the Kaplan–Meier method and log-rank tests. The optimal cutoff values for gene expression were determined using the survminer package in R. The tumor mutation burden analysis for OSCC patients in high and low m1A score clusters was performed using the maftools package in R. Spearman correlation analysis was performed to calculate the correlation coefficient of gene expression and correlation between m1A score and known biological gene signatures using previously reported gene sets [8,9]. Student’s *t*-test was used to estimate the statistical differences between two groups, while one-way ANOVA and Kruskal–Wallis tests were performed when more than two groups were considered. In all cases, the *p*-value was two-sided, with *p* < 0.05 considered to be statistically significant.

## Data Availability

All data in this study are available in TCGA and GEO datasets.

## Supplementary Materials

The following are available online at https://www.mdpi.com/article/10.3390/ijms221910302/s1, Figure S1: Survival analysis of m1A regulators in patients with oral squamous cell carcinoma in the validation cohort. Figure S2: Unsupervised consensus clustering of 10 m1A regulators in patients with oral squamous cell carcinoma. Figure S3: Heatmap showing the activation states of biological pathways using GSVA enrichment analysis. Figure S4: Unsupervised clustering of 827 m1A phenotype–related genes in patients with oral squamous cell carcinoma. Table S1: Summary of m1A modification-related differentially expressed genes.
