# Supplementary material for: The Impact of m1A Methylation Modification Patterns on Tumor Immune Microenvironment and Prognosis in Oral Squamous Cell Carcinoma
Source: Int J Mol Sci. 2021 Sep 24;22(19):10302. doi: 10.3390/ijms221910302 (PMC8508946; doi:10.3390/ijms221910302)
Supplement: Supplementary file 1 [file ijms-22-10302-s001.zip › Supplementary_Information.pdf]

m1A methylation modification patterns impact tumor immune microenvironment and prognosis in oral squamous cell carcinoma

Supplementary Information

Figure S1 Survival analysis of m1A regulators in patients with oral squamous cell carcinoma in the validation cohort.

Figure S2 Unsupervised consensus clustering of 10 m1A regulators in patients with oral squamous cell carcinoma. **(a)-(g)** Unsupervised consensus clustering of 405 patients with oral squamous cell carcinoma. Consensus matrices of the training cohort for k = 2 – 4 (a-d), cumulative distribution function (CDF) (e), the relative change in area under the CDF curve (g), and tracking plot (g) for k = 2 to 9. **(h)-(n)** Unsupervised consensus clustering of 97 patients in the validation cohort. Consensus matrices of the training cohort for k = 2 – 4 (h-k), cumulative distribution function (CDF) (l), relative change in area under the CDF curve (m), and tracking plot (n) for k = 2 to 9. (**o**) Expression of 10 m1A methylation regulators between two m1A modification patterns in the validation cohort. *P < 0.05, ***P < 0.001).

Figure S3 The heatmap shows the activation states of biological pathways using GSVA enrichment analysis. **(a)** m1A cluster A vs m1A cluster B; **(b)** m1A cluster B vs m1A cluster C.

Figure S4 Unsupervised clustering of 827 m1A phenotype-related genes in patients with oral squamous cell carcinoma. **(a)** Venn diagram of 827 m1A phenotype-related genes. **(b)**-**(h)** Unsupervised consensus clustering of 405 patients with oral squamous cell carcinoma. Consensus matrices of the training cohort for k = 2 – 4 (b-e), cumulative distribution function (CDF) (f), the relative change in area under the CDF curve (g), and tracking plot (h) for k = 2 to 9. **(i)-(o)** Unsupervised consensus clustering of 97 patients in the validation cohort. Consensus matrices of the training cohort for k = 2 – 4 (i-l), cumulative distribution function (CDF) (m), relative change in area under the CDF curve (n), and tracking plot (o) for k = 2 to 9.
