# Supplementary figures and images for: The Impact of m1A Methylation Modification Patterns on Tumor Immune Microenvironment and Prognosis in Oral Squamous Cell Carcinoma
Source: Int J Mol Sci. 2021 Sep 24;22(19):10302. doi: 10.3390/ijms221910302 (PMC8508946; doi:10.3390/ijms221910302)

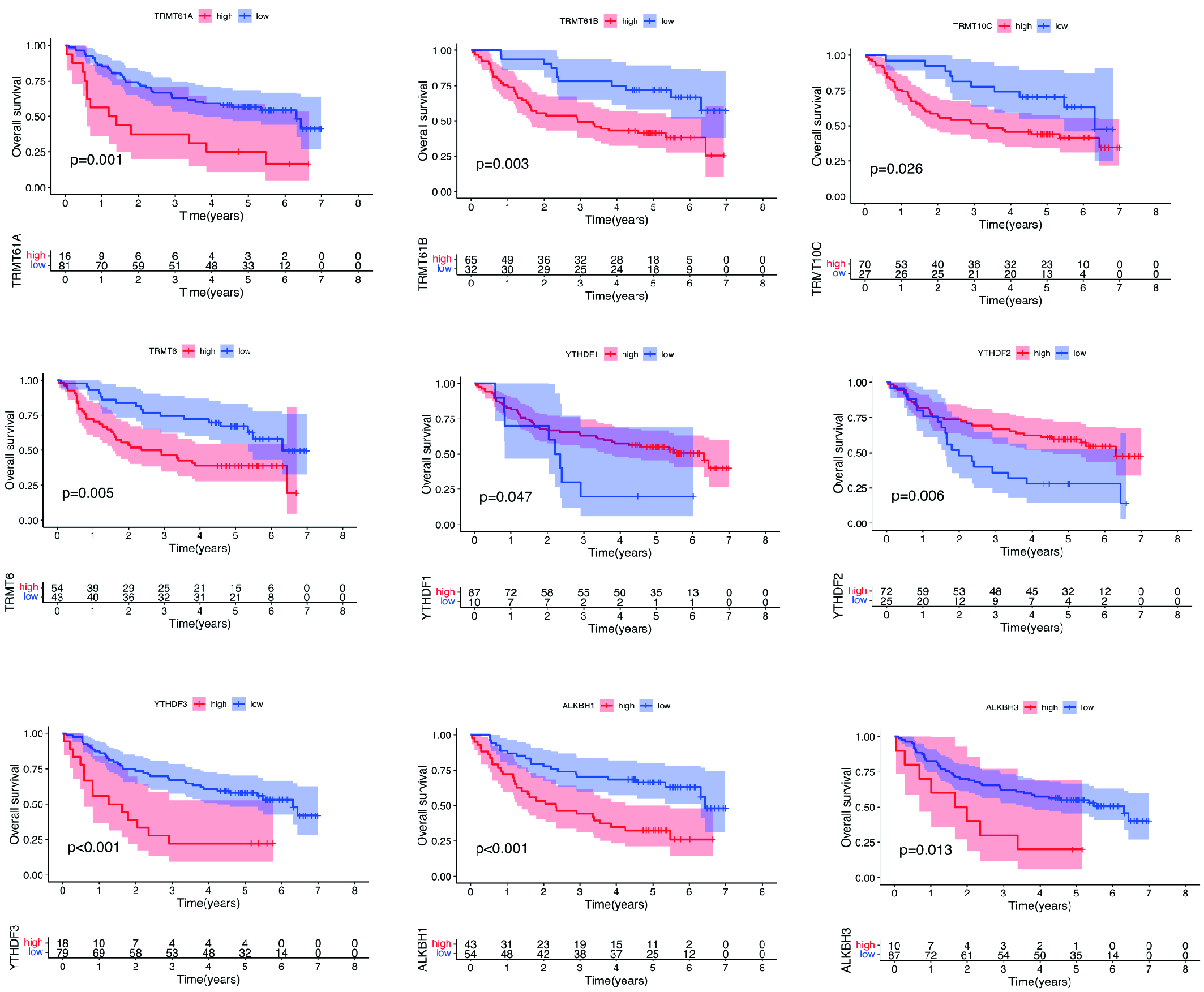

Supplement: Supplementary file 1 [file ijms-22-10302-s001.zip › S F1.jpg]

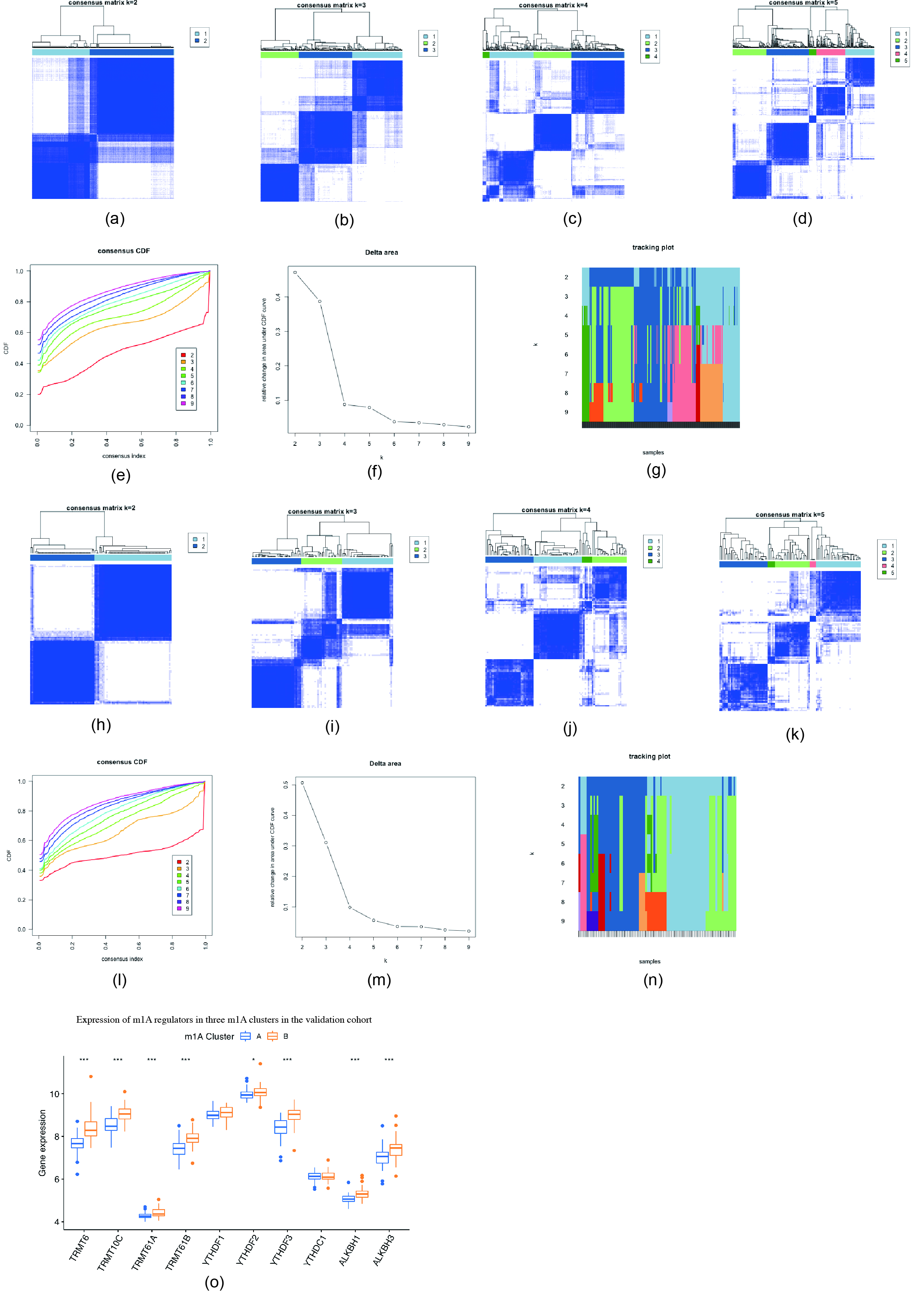

Supplement: Supplementary file 1 [file ijms-22-10302-s001.zip › S F2.jpg]

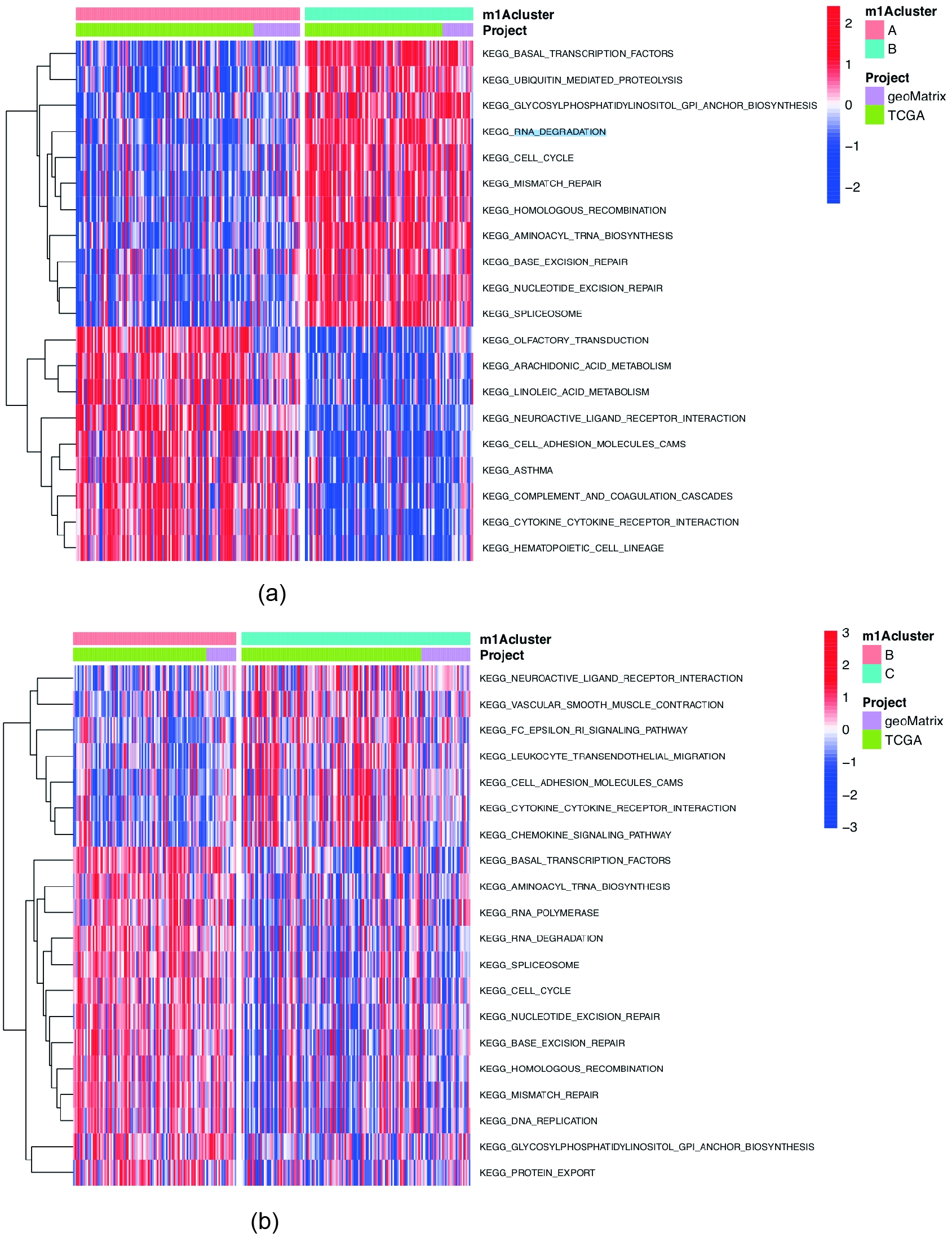

Supplement: Supplementary file 1 [file ijms-22-10302-s001.zip › S F3.jpg]

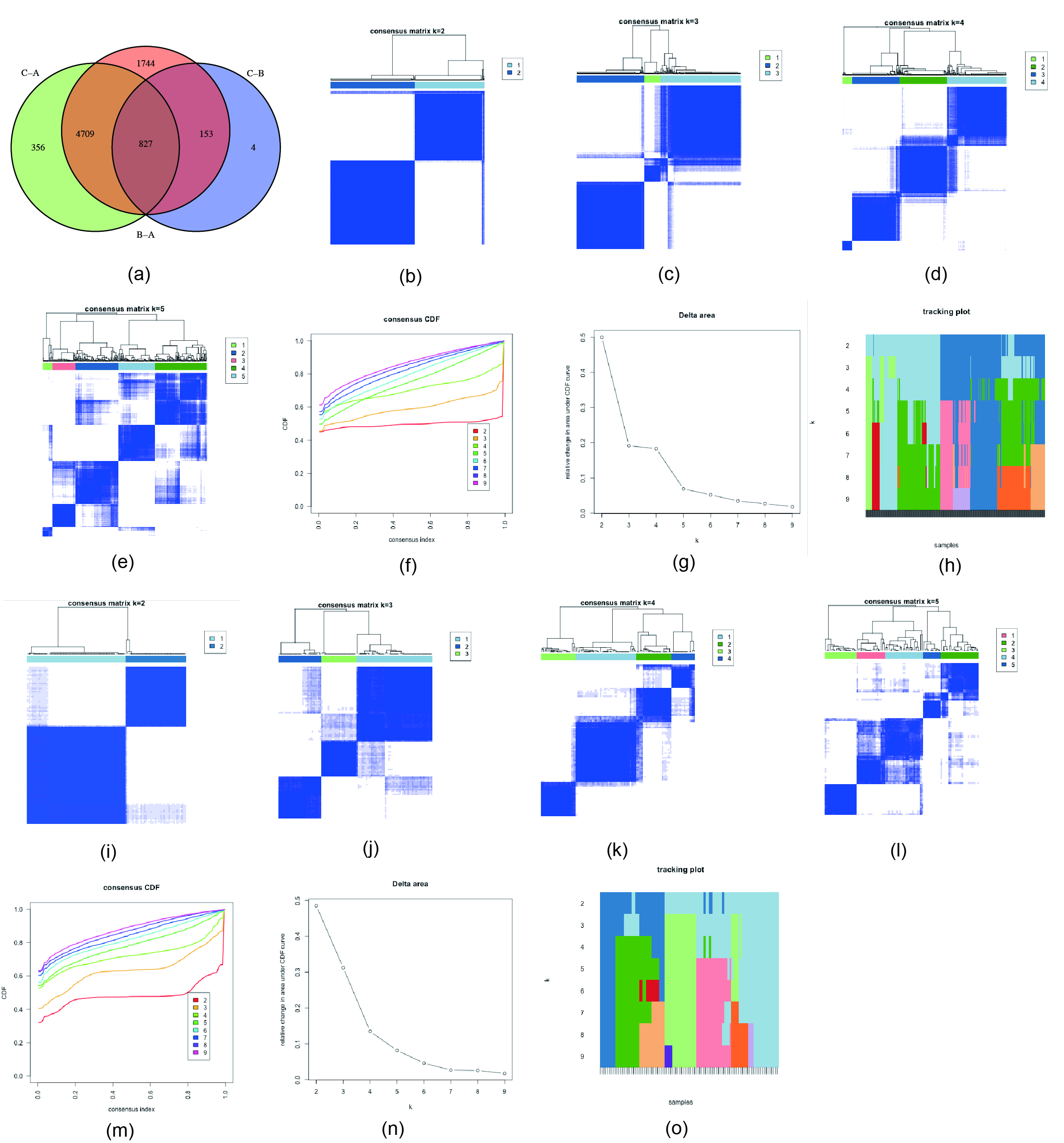

Supplement: Supplementary file 1 [file ijms-22-10302-s001.zip › S F4.jpg]
